# Quantitative 3D Characterization for Kinetics of Corrosion Initiation and Propagation in Additively Manufactured Austenitic Stainless Steel

**DOI:** 10.1002/advs.202201162

**Published:** 2022-10-26

**Authors:** Jianli Li, Anthony E. Hughes, Y. S. Yang, Majid Laleh, Haipeng Wang, Xufang Zhang, Jie Ma, Wei Xu, Mike Y. Tan

**Affiliations:** ^1^ Institute of Theoretical Physics School of Physics and Electronic Engineering and Collaborative Innovation Center of Extreme Optics Shanxi University Taiyuan Shanxi 030006 China; ^2^ Institute for Frontier Materials Deakin University Waurn Ponds VIC 3216 Australia; ^3^ Commonwealth Scientific and Industrial Research Organisation (CSIRO) Private Bag 10 Clayton South VIC 3169 Australia; ^4^ School of Engineering Deakin University Waurn Ponds VIC 3216 Australia; ^5^ State Key Laboratory of Quantum Optics and Quantum Optics Devices Institute of Laser Spectroscopy and Collaborative Innovation Center of Extreme Optics Shanxi University Taiyuan Shanxi 030006 China

**Keywords:** additive manufacturing, austenitic stainless steel, corrosion kinetics, data‐constrained modeling, in situ X‐ray CT

## Abstract

In situ X‐ray computed tomography (X‐ray CT) is used to investigate the effects of characteristic microstructural features on the pitting initiation and propagation in austenitic stainless steel specimens prepared with laser powder bed fusion (LPBF) additive manufacturing. In situ X‐ray CT in probing the mechanism and kinetics of localized corrosion is demonstrated by immersing two LPBF specimens with different porosities in an aggressive ferric chloride solution for the evaluation of corrosion. X‐ray CT images are acquired from the specimens after every 8 hours of immersion over an extended period of time (216 hours). Corrosion pit growth is then quantitatively analyzed with a data‐constrained modeling method. The pitting growth mechanism of LPBF stainless steel is found to be different from that of conventional stainless steels. More specifically, the mechanism of corrosion pit initiation is closely correlated with the original lack of fusion porosity (LOF) distribution on the surface of the specimens and preferential pit propagation through the LOF pores inside the specimens. Pit growth kinetics are derived from pit volume changes determined through 3D data analysis. The pit growth kinetics in LPBF specimens are found to vary in the initial pit formation, competitive pit propagation, and the dominant pit growth stages.

## Graphical abstract

There are two classes of corrosion pits in the LPBF 316L stainless steel. One does not change significantly with time after initial formation while the other one whose volumes increase with time. The propagation of pitting is closely related to porosity microstructures in the sample. The corrosion pit growth versus time has an approximate power‐law behavior.

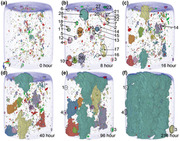

## 1 Introduction

Additive manufacturing (AM) refers to any process of making 3D objects from a digital design in a layer‐upon‐layer manner. Some metal AM processes use either a laser/electron beam, electric arc, or gas plasma as a heating source and powder/wire/sheet as the feeding material.^[^ ^1^ ^]^ Such manufacturing processes are associated with a sequence of rapid heating and cooling cycles along with large temperature gradients,^[^ ^2^, ^3^ ^]^ leading to distinct microstructural features in terms of porosity,^[^ ^4^ ^]^ grain morphology,^[^ ^5^ ^]^ subgrain structure,^[^ ^6^ ^]^ grain boundary character,^[^ ^7^ ^]^ phase constituents,^[^ ^8^ ^]^ and precipitates/inclusions^[^ ^9^, ^10^ ^]^ compared to those of conventionally manufactured material. These unique and complex microstructures are known to be closely linked to the corrosion characteristics of AM stainless steels.^[^ ^11^, ^12^, ^13^, ^14^, ^15^ ^]^

Austenitic stainless steels produced by laser powder bed fusion (LPBF) AM technology generally have higher pitting corrosion resistance than their conventionally manufactured counterparts, due to the absence of detrimental MnS inclusions as the most susceptible pit initiation sites.^[^ ^12^, ^13^, ^14^, ^15^ ^]^ However, the presence of process‐induced porosity in the metal AM materials,^[^ ^16^, ^17^, ^18^ ^]^ particularly lack‐of‐fusion (LOF) pores, leads to weaker pitting corrosion resistance and re‐passivation ability in LPBF stainless steels.^[^ ^19^, ^20^ ^]^ In general, specimens with low densities (typically <99%) show pitting potentials well below (at least 250 mV) those of the high‐density ones and in some cases even below that of the conventionally manufactured austenitic stainless steels based on polarization measurements.

In recent study by the authors, it was found that the localized corrosion resistance of the LPBF 316L stainless steel (hereafter 316L SS) can differ widely, depending on the density and type of the pores in the LPBF specimens.^[^ ^19^ ^]^ The LOF pores have been shown to have a detrimental effect on both corrosion initiation and propagation as determined through polarization studies supported by 2D and 3D analyses. Similar conclusions were drawn by Trelewicz et al.^[^ ^21^ ^]^ for the decreased corrosion resistance of LPBF 316L SS in a 0.1 m HCl solution and by Schaller et al.^[^ ^22^ ^]^ for the LPBF 304L SS in a 0.6 m NaCl solution. Effects of other microstructural features on corrosion have also been reported. For instance, Prieto et al.^[^ ^23^ ^]^ showed a corrosion rate almost five times higher for the LPBF 316L SS compared to its as‐received conventional counterpart (10 mm year^−1^ vs 2 mm year^−1^) in a 6 wt% ferric chloride solution at 55 °C for 48 h, which was attributed to the higher susceptibility of melt pool boundaries to pitting corrosion in the AM material. Similarly, Cruz et al.^[^ ^24^ ^]^ reported a weight loss of almost 4 mg cm^−2^ d^−1^ (≈2 mm year^−1^) for LPBF 316L SS after 72 h exposure to 6 wt% ferric chloride solution at ambient temperatures. All these results confirm the importance of alloy microstructure around pores in the corrosion behavior of AM materials when exposed to aggressive environments. Such influence becomes even more important considering the fact that suppressing pore formation is still a challenge in metal AM,^[^ ^3^, ^25^, ^26^ ^]^ especially in producing complex geometries where controlling the melt pool characteristics is difficult at specific locations like sharp edges, where the possibility of pore formation increases. Apart from the geometrical considerations of pores, it has recently been shown by the authors that pores, in particular LOF pores, might not be considered as simple voids as they are partially covered by oxide/silicate films that develop during melting/solidification in metal AM processing.^[^ ^4^ ^]^ This is expected to influence the materials properties and corrosion performance. Moreover, as demonstrated in the authors' previous work, LOF pores can persist to high densities (>99%) and their influence on the microstructure around them in the powder bed plain is only beginning to be studied.

Despite progress made to date, the details of the kinetics of corrosion propagation within these LOF microstructures in AM stainless steels have not yet been examined. This work aims to understand how the unique microstructural features in typical AM 316L SS specimens affect the corrosion, from initiation to propagation with a particular focus on LOF microstructures. The approach presented in this paper uses X‐ray computed tomography (X‐ray CT) to study in situ 3D mapping of porosity and pit growth and follows on from a number of X‐ray CT studies that examine corrosion propagation in steels,^[^ ^27^, ^28^, ^29^, ^30^, ^31^ ^]^ and corrosion inhibitor migration in metal coatings.^[^ ^32^ ^]^ Here, two LPBF 316L SS specimens with different levels of porosity were selected and corrosion was followed as a function of exposure time to a ferric chloride solution. For the first time the kinetics of pit volume and surface area growth in steels are determined both globally and for individual pits. This approach reveals the interplay between individual and global pit growth particularly when pits become larger and compete for cathodic current.

## 2 Experimental Section

### 2.1 Sample Preparation

Argon gas‐atomized 316L SS spherical powder of 10–45 µm (SLM Solutions) was used as feeding material for LPBF processing. Cubes of 10 × 10 × 10 mm^3^ were produced using an SLM machine (SLM125‐SLM Solutions Group AG). Two specimens with different levels of porosity were produced by changing laser power, laser scanning speed, and hatch spacing, while other processing parameters were kept constant (**Table** **1**). LPBF processing was conducted under an argon atmosphere (oxygen content was kept below 100 ppm) to reduce the possibility of oxidation reactions. The build plate was preheated to 200 °C to reduce the thermal gradients between the part and substrate and hence establishment of residual stresses. A meander hatch pattern with 67° rotation between successive layers was used as the scanning strategy for LPBF processing. After completion of the building process, the specimens were cooled down and removed from the build plate by wire‐cutting.

**Table 1:** Processing parameters for production of LPBF specimens along with density values based on the Archimedes principles measurements

Specimen	Laser power [W]	Scanning speed [mm s^−1^]	Hatch spacing [µm]	Layer thickness [µm]	Density [%]
Mid‐density	125	600	80	30	99.05 ± 0.06
Low‐density	150	800	100	30	98.77 ± 0.08

Densities of the LPBF specimens were measured based on the Archimedes principle using a Densimeter (Electronic Densimeter, SD‐200L) with an accuracy of 0.1 mg cm^−3^. Density measurements were repeated five times for each specimen and the average values are reported in Table 1. The standard deviation was quoted in the table as error bars. Prior to density measurements, the specimens were ultrasonically cleaned in ethanol for 10 min to degrease them and also to remove the residual powder from the specimens’ surfaces, followed by drying in hot air. For simplicity, the specimens with densities of 98.77% and 99.05% are labeled as low‐ and mid‐density specimens, respectively. These specimens were selected for this study because they were expected to be sensitive to pitting based on the observations in authors’ previous paper,^[^ ^19^ ^]^ thus enabling monitoring of the corrosion pit propagation kinetics as the main focus of this work.

**Figure 1:**
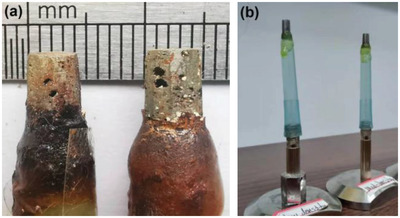
LPBF specimens. a) After corrosion, the left is the low‐density and the right is the mid‐density specimen. b) Before corrosion, the left is the low‐density and the right is the mid‐density specimen.

Two pieces with 5 × 5 × 10 mm^3^ were cutout from the cubes using an electrical discharge wire cutting machine, then ground to ≈4–5 mm diameter cylindrical specimens, as shown in **Figure** **1**a. For the mid‐density specimen, the cylindrical specimen axis was parallel to the build direction during LPBF processing. For the low‐density specimen, it was parallel to the powder‐bed plane (perpendicular to the building direction during LPBF processing). The specimens were then mounted on polycarbonate tubes using an epoxy resin. They were attached to sample holders which were designed for convenient repositionable mounting on the X‐ray imaging sample stage, as shown in Figure 1b.

### 2.2 Corrosion Experiments

A 6 wt% ferric chloride solution was used as the corrosive medium, which was prepared by dissolving 60 g of ferric chloride in 940 g of purified water in a glass beaker. While this is an aggressive test solution, it is a standard test solution for evaluating pitting and crevice corrosion resistance of stainless steels (ASTM‐G48‐11, https://www.astm.org/standards/g48). It may also facilitate the onset of salt film formation given the high iron and chloride ion concentration, noting that FeCl_2_ (not FeCl_3_) was observed in the salt film.^[^ ^33^ ^]^ A set of X‐ray CT projection images was acquired before the corrosion experiment. The immersion experiments were conducted in 27 time intervals for a total corrosion exposure time of 216 h. At each time interval, the specimens were hung upside down and immersed into the solution at room temperature (24 ± 2 °C) for 8 h. The ferric chloride solution was stirred with a stir bar before the specimens were immersed at each step. After each immersion step, the specimens were removed from the solution, dried with absorbent cotton, and placed back on the CT sample stage at the same initial angular position as before corrosion and all previous corrosion experiments. In this fashion all datasets had the same reference position. This approach inevitably means that there were potentially two relevant time scales at each step: i) immersion time and ii) immersion time plus CT imaging time. In the latter case, upon removal of the specimens from corrosion experiment some FeCl_3_ solution drained from some pits but there may have been traces of solution inside the corrosion pits. Therefore, the corrosion reaction should be slowed down such that the microstructure is consistent during X‐ray CT imaging, but it may not stop completely. In this paper the results are interpreted per the immersion time.

### 2.3 Time‐Lapsed X‐Ray CT Imaging

The X‐ray projection images of the LPBF 316L SS specimens were acquired using a Sanying Precision Engineering nanoVoxel‐3000H X‐ray microfocus CT machine. The X‐ray tube source voltage and current were set at 210 kV and 50 µA, respectively. A 1 mm thick Cu filter was mounted on the X‐ray tube to reduce the beam‐hardening effect.^[^ ^34^ ^]^ The projection images were captured using a flat‐panel detector with a native pixel size of 127 µm. With a source‐to‐object distance (SOD) of 17.56 mm and a source‐to‐detector distance (SDD) of 557.20 mm, an effective pixel size of 4 µm was achieved. Each projection image was acquired with an exposure time of 1.2 s. To reduce the photon‐counting noise, four projection images were averaged to generate a combined projection image at each sample rotation angle. A total of 1440 background‐corrected combined projection images were collected for each dataset. The X‐ray CT slices were reconstructed from the projection images using the Sanying VoxelStudio software with reconstruction parameters optimized for further reduction of the beam‐hardening artifact. Using the same parameters as above, this X‐ray CT imaging and reconstruction process was repeated after each corrosion experiment steps for each specimen. For each specimen, a total of 28 datasets were accumulated.

**Figure 2:**
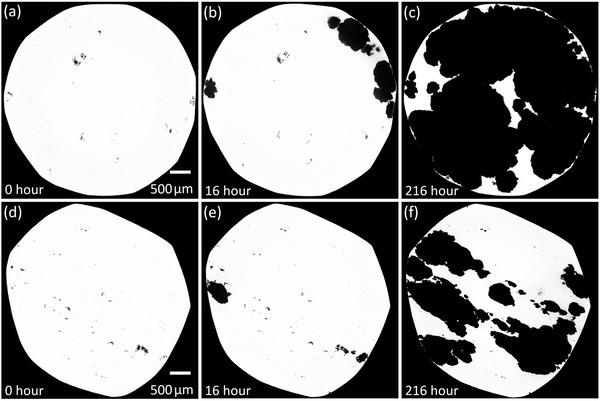
Typical X‐ray CT slices of the original specimens and after different immersion times. The display intensity of a pixel is proportional to its X‐ray total linear absorption coefficient. Each image has 1181 × 1181 pixels. A pixel represents a physical size of 4 µm × 4 µm. a–c) The 1300th CT slices for the mid‐density specimen. d–f) The 1200th CT slices for the low‐density specimen.

Typical CT reconstructed slices are shown in **Figure** **2**. The CT slices have two dominant intensity levels: the bright areas correspond to the metal (316L SS) and the dark areas correspond to either the pre‐existing porosity or corrosion pits. For each specimen, 20 equally spaced CT slices were selected to estimate the CT reconstructed X‐ray linear absorption coefficients for pure metal and corrosion pits across all specimens (2.20 and 0.57 cm^−1^ for pure 316L SS and pits, respectively). A nonzero value for the pits is primarily related to the fact that the pits were filled with a mixture of ferric chloride solution and the corrosion product after the specimen was taken out of the solution and dried. Another factor for such nonzero value was the point spread function (PSF) effect.^[^ ^35^ ^]^

### 2.4 DCM Microstructural Analysis

The data‐constrained modeling (DCM) nonlinear optimization method^[^ ^36^ ^]^ was used to obtain porosities in each voxel before corrosion. The porosities of the mid‐ and low‐density samples were calculated as 0.971% and 1.22%, respectively, which are consistent with densities determined using Archimedes principle as detailed in the previous section. That is, the two samples should be reasonable representations of the bulk mid‐ and low‐density samples. For the corroded specimens, the sizes of corrosion pits (typically millions of cubic microns) were much larger than both the voxel size itself (4 µm × 4 µm × 4 µm = 64 µm^3^) and the number of partially porous voxels containing both metal and pore at the metal–pore interface. A more CPU‐efficient DCM discrete‐least‐squares segmentation method^[^ ^36^, ^37^ ^]^ was used to segment the pore distributions for the corroded specimens, without any detectable decrease in numerical accuracy.

The DCM analysis was performed within the sample geometry. For the purpose of quantitative comparisons, for each sample, the same boundaries were used before and after different immersion times. The reference boundaries of the mid‐ and low‐density specimens before corrosion were established in the DCM software in reference to their linear absorption coefficient pixel values on the CT slices. For corroded specimens, the spatial and angular positions of the reference boundaries were fine‐tuned to compensate for the minor sample repositioning error on the X‐ray CT imaging sample stage.

## 3 Results

### 3.1 Distribution of Pores and Corrosion Pits

**Figure 3:**
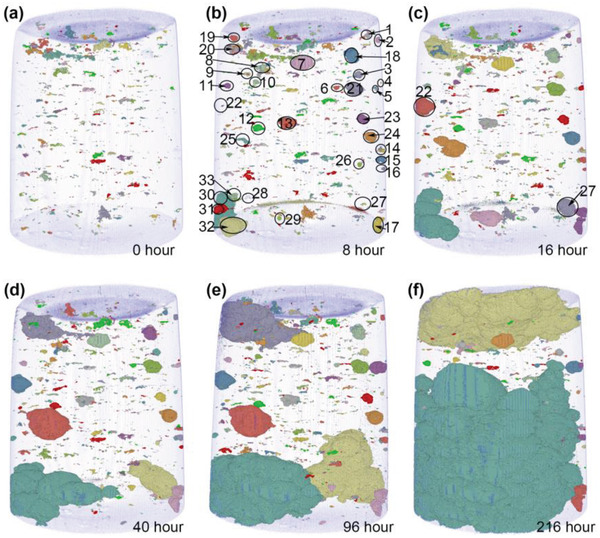
3D pore clusters and pits of the mid‐density 316L SS specimen a) prior to corrosion and b–f) after different immersion times. Voxels in the same cluster were displayed with the same color. The remaining volume is the 316L stainless steel. 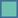 1st, 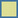 2nd, 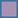 3rd, 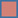 4th largest clusters.

**Figure 4:**
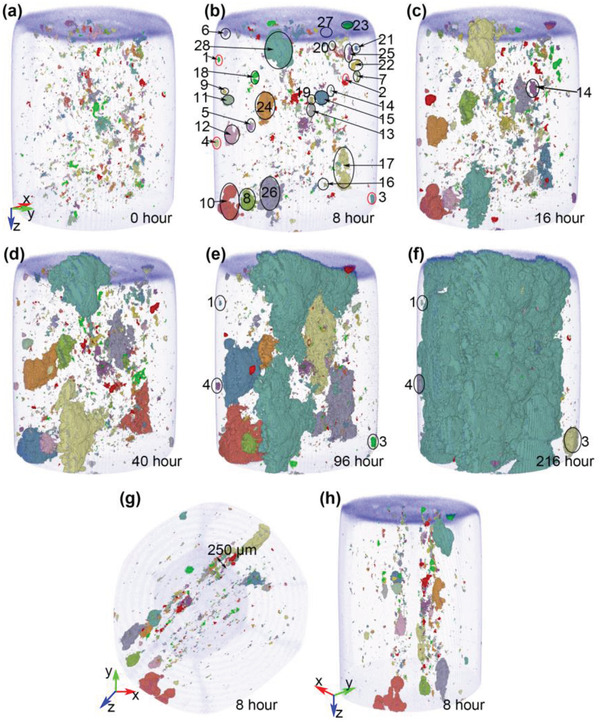
3D pore clusters of the low‐density 316L SS specimen before corrosion and after different immersion time. Voxels in the same cluster were displayed as the same color. The remaining volume is the 316L SS metal. (a)–(f) have the same viewing angle which is partially side‐on. 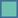 1st,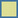 2nd,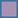 3rd,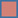 4th largest clusters. Viewing angles for (g) and (h) are adjusted to highlight internal anisotropy.

**Figure** **3** shows the distribution of pores and pits as determined through 3D pore clustering of the mid‐density specimen prior to corrosion (a) and after different immersion times (b–f). In these images, two neighboring voxels were regarded as connected when they had pore volume fractions above 0.5. Such connected voxels form pore clusters.^[^ ^36^, ^38^, ^39^ ^]^ After each immersion, the volume of individual pits were determined by counting the number of voxels (voxel = 64 µm^3^); these are presented in **Tables** **2** and **3** for the mid‐ and low‐density specimens, respectively. The pore cluster volumes at time zero (i.e., LOF structures) were calculated in the specimen regions at the sites of the first detected corrosion pits. The DCM datasets are available for download.^[^ ^40^ ^]^ Numerical labels were assigned to the 33 relevant corrosion pit locations in the mid‐density specimen (Figure 3b) and 28 pits in the low‐density specimen (**Figure** **4**b).

**Table 2:** Corrosion pit volumes of the mid‐density specimen versus immersion time (IT) for cluster labels 1–33 in Figure 3b. Bold (blue background) represents time where pits ceased to grow, and italics (gray background) represent merged pits

	Corrosion pit volume (×10^−3^ mm^3^) for different pit labels																																
IT [h]	1	2	3	4	5	6	7	8	9	10	11	12	13	14	15	16	17	18	19	20	21	22	23	24	25	26	27	28	29	30	31	32	33
0	0.031	0.020	0.322	0	0	0.045	0.220	0.139	0.131	0.043	0.365	0.399	1.984	1.225	0.498	0.074	2.436	10.64	0.017	3.546	0.406	0.660	0.930	0.102	1.857	0.049	1.494	2.470	0.091	1.991	0.720	1.153	3.057
8	**1.616**	**1.715**	**6.653**	0.021	**0.085**	**0.630**	**16.18**	**1.463**	**2.324**	**3.082**	**3.093**	**9.198**	**36.89**	**2.470**	**5.452**	**0.632**	13.21	**26.29**	4.853	15.19	**54.06**	0.660	**14.79**	20.56	2.623	**1.873**	1.494	2.470	0.304	196.5	11.68	70.96	46.66
16	1.426	1.552	6.450	**1.368**	0.098	0.554	16.09	1.373	2.156	2.918	2.886	8.981	34.80	2.247	5.099	0.540	26.77	25.10	*194.8*		53.23	**59.69**	14.56	**57.36**	53.88	1.716	73.65	2.470	39.26	*637.2*			
24	1.556	1.721	6.583	1.393	0.099	0.606	16.25	1.439	2.184	2.940	2.893	9.020	36.58	2.433	5.432	0.617	27.09	25.56	*295.7*		53.68	60.46	14.68	57.81	**216.6**	1.793	155.0	2.470	168.0	*790.1*			
32	1.542	1.722	6.556	1.388	0.100	0.606	16.46	1.472	2.290	3.059	2.974	9.180	36.48	2.522	5.407	0.611	**43.23**	25.52	*342.6*		53.14	60.68	14.65	57.53	218.3	1.782	471.5	2.470	271.5	*913.3*			
40	1.465	1.669	6.323	1.352	0.097	0.576	16.14	1.399	2.209	2.979	2.865	9.010	36.61	2.532	5.233	0.581	42.75	24.91	*398.4*		52.73	60.23	14.20	57.29	218.4	1.752	603.9	2.470	*1728.6*				
48	1.536	1.711	6.634	1.387	0.107	0.600	16.29	1.388	2.221	2.996	2.912	9.087	36.96	2.554	5.482	0.624	43.83	25.75	*458.6*		53.82	60.76	14.82	58.12	219.8	1.828	784.0	2.470	*2104.1*				
56	1.542	1.717	6.682	1.384	0.101	0.611	16.38	1.448	2.278	3.079	2.971	9.234	37.29	2.438	5.344	0.607	44.11	25.99	*529.2*		53.86	60.91	14.90	58.05	221.0	1.805	1178.5	2.470	*2803.2*				
64	1.550	1.716	6.566	1.417	**34.65**	0.618	16.31	1.487	2.191	2.933	2.945	8.910	36.32	2.588	5.488	0.634	44.33	25.50	*644.3*		53.65	60.32	14.65	57.70	218.1	1.894	1807.1	43.50	*3129.6*				
72	1.446	1.608	6.489	1.348	34.85	0.587	16.09	1.445	2.271	3.010	3.001	9.027	36.60	2.398	5.153	0.556	43.73	24.96	*868.8*		53.24	60.57	14.54	57.10	219.2	*2301.6*		*3475.2*					
80	1.460	1.544	6.397	1.313	34.47	0.571	15.84	1.369	2.199	2.881	2.933	8.890	36.41	2.342	5.139	0.568	43.01	25.01	*1067.3*		53.33	59.89	14.35	57.00	217.7	*2750.1*		*4118.2*					
88	1.442	1.653	6.592	1.380	35.40	0.601	16.37	1.455	2.339	3.109	2.996	9.223	37.13	2.614	5.516	0.619	44.02	25.34	*1529.6*		53.43	61.13	14.77	58.07	220.5	*2924.80*		*4780.8*					
96	1.481	1.671	6.577	1.396	35.27	0.617	16.65	1.551	2.295	3.018	2.980	8.889	36.63	2.537	5.597	0.659	44.32	25.07	*1993.3*		53.39	60.98	14.77	58.11	221.4	*3366.40*		*5209.6*					
104	1.440	1.637	6.502	1.363	35.10	0.591	16.38	1.468	2.245	2.914	2.908	8.718	36.41	2.585	5.558	0.641	43.39	24.62	*2370.3*		53.22	60.70	14.67	58.01	219.9	*3366.40*		*5753.6*					
112	1.463	1.557	6.596	1.340	34.96	0.589	16.01	1.431	2.384	3.020	3.074	9.088	36.79	2.644	5.554	0.647	43.58	25.06	*2661.4*		53.72	60.46	14.76	57.93	219.3	*4006.40*		*6246.4*					
120	1.380	1.509	6.473	1.294	34.57	0.546	15.92	1.449	2.431	3.078	3.055	9.098	36.50	2.329	5.369	0.603	42.65	24.56	*3320.2*		53.26	60.39	14.41	57.22	219.1	*4044.80*		*7104.0*					
128	1.342	1.483	6.481	1.280	34.85	0.531	15.82	1.311	2.340	3.043	2.824	9.086	36.60	2.336	5.330	0.585	42.49	24.32	*3890.4*		52.92	60.25	14.42	57.35	217.9	*4358.40*		*7744.0*					
136	1.340	1.547	6.425	1.328	34.59	0.574	16.05	1.400	2.546	3.085	2.954	9.147	36.72	2.614	5.589	0.647	43.22	24.63	*4643.1*		52.86	59.85	14.21	58.46	293.8	*4358.40*		*8192.0*					
144	1.241	1.284	6.454	1.206	34.42	0.544	15.82	1.376	2.451	2.905	3.072	8.970	36.45	2.291	5.178	0.566	42.10	23.78	*5261.2*		53.18	60.42	14.54	58.09	295.1	*4537.60*		*9024.0*					
152	1.368	1.509	6.454	1.293	34.32	0.577	15.77	1.431	2.508	3.014	2.969	9.088	36.60	2.366	5.540	0.635	42.71	24.74	*5858.6*		52.69	61.42	14.44	58.04	294.6	*4966.40*		*9920.0*					
160	1.387	1.429	6.498	1.301	34.46	0.565	15.70	1.377	2.595	3.121	3.034	8.989	36.60	2.576	5.605	0.617	42.69	25.08	*6257.5*		53.38	60.24	14.52	58.58	*16768.0*								
168	1.341	1.383	6.470	1.246	34.18	0.552	15.76	1.361	2.417	2.937	2.882	8.705	35.93	2.551	4.363	0.615	42.53	24.63	*6421.0*		52.90	59.48	14.53	58.68	*18752.0*								
176	1.342	1.415	6.357	1.262	34.10	0.553	15.79	1.293	2.353	2.839	2.828	8.602	35.48	2.603	4.373	0.650	42.19	24.71	*6753.3*		52.78	59.02	14.80	*22272.0*									
184	1.430	1.445	6.594	1.293	34.39	0.570	15.86	1.421	2.493	2.958	3.013	8.804	36.38	2.855	4.415	0.751	43.93	25.71	*6999.9*		53.65	59.64	*24256.0*										
192	1.351	1.455	6.375	1.283	34.17	0.526	15.95	1.404	2.554	3.045	2.944	8.957	36.82	2.644	4.450	0.668	43.05	24.61	*7263.8*		52.67	59.68	*25536.0*										
200	1.279	1.288	6.273	1.176	33.37	0.497	15.44	1.316	2.533	2.997	2.847	8.813	36.13	2.432	4.276	0.606	41.78	23.81	*7992.9*		51.80	*27059.5*											
208	1.313	1.374	6.432	1.245	34.38	0.520	15.58	1.268	2.530	3.044	2.853	8.943	36.31	2.480	4.376	0.619	41.99	24.07	*8433.0*		*29443.5*												
216	1.372	1.492	6.610	1.285	34.63	0.558	15.92	1.364	2.531	3.133	2.922	9.127	514.9	2.630	4.461	0.632	42.30	*9161.8*			*31177.8*												

**Table 3:** Corrosion pit volumes of the low‐density 316L SS specimen versus immersion time (IT) for the labeled clusters in Figure 4b. Italics (gray background) indicates merged pits, and bold (blue background) indicates the time small pits ceased growing

IT [h]	Corrosion pit volume (×10^−3^ mm^3^) for different pit labels																												
	1	2	3	4	29	5	6	7	8	9	10	11	12	13	14	15	16	17	18	19	20	21	22	23	24	25	26	27	28
0	0.0003	0.018	0.010	0.025		0.016	0.158	0.399	0.725	0.002	2.630	0.019	0.399	0.478	1.798	1.400	1.160	16.26	0.144	0.024	0.048	0.300	0.767	0.313	6.351	1.235	7.395	0.380	6.001
8	0.285	0.728	3.222	1.756		1.304	0.624	1.317	19.28	0.529	66.35	7.621	17.18	13.16	1.798	59.04	**19.39**	91.35	1.865	2.238	1.213	0.907	5.785	4.471	32.75	2.289	70.92	0.380	111.6
16	**0.440**	**0.966**	**3.661**	2.165		1.542	0.826	1.729	21.85	0.677	144.6	8.482	88.54	14.47	14.73	173.0	19.39	120.3	**2.447**	2.454	1.377	1.053	6.809	4.970	38.12	2.729	207.1	0.380	198.5
24	0.444	1.026	3.752	2.175		1.548	0.858	1.931	22.21	0.670	169.7	8.498	131.1	14.76	**15.01**	225.9	*165.8*		2.485	**2.533**	**1.477**	**1.151**	**7.116**	5.240	38.57	3.078	352.9	0.380	379.6
32	0.446	0.989	3.725	2.180		1.563	0.888	1.813	22.22	0.673	214.7	8.513	192.0	14.68	14.97	358.0	*260.4*		2.517	2.511	1.442	1.052	6.862	5.416	38.64	2.838	414.9	0.380	547.4
40	0.475	1.004	3.776	**2.198**		**1.615**	**0.941**	**1.940**	**22.44**	**0.695**	309.9	**8.611**	240.4	**14.84**	*462.9*		*318.8*		2.590	2.560	1.463	1.133	7.058	**5.693**	**38.74**	**3.045**	483.2	0.380	643.3
48	0.386	0.937	3.562	1.998		1.481	0.790	1.716	21.50	0.597	340.6	8.158	259.0	14.08	*584.1*		*430.5*		2.319	2.376	1.378	1.012	6.560	5.373	36.67	2.741	608.8	10.38	751.0
56	0.419	0.796	3.584	1.998		1.387	0.874	1.443	21.40	0.604	382.6	8.164	283.0	13.78	*718.5*		*547.8*		2.136	2.241	1.248	0.865	5.880	4.777	35.59	2.185	731.3	38.59	927.6
64	0.547	0.936	4.546	2.507		1.570	1.046	1.764	22.58	0.716	424.7	8.706	319.2	14.92	*968.1*		*723.2*		2.507	2.533	1.370	1.018	6.635	5.550	37.72	2.664	819.3	*1297.3*	
72	0.334	0.997	3.620	1.938		1.310	0.654	1.904	22.22	0.533	480.5	8.128	387.2	15.02	*1252.0*		*922.3*		2.194	2.523	1.437	1.117	7.057	4.365	37.54	9.976	898.3	*1646.3*	
80	0.432	0.933	4.471	2.070		1.441	0.868	1.623	21.60	0.619	543.0	8.232	428.2	14.41	*1443.6*		*1072.3*		2.279	2.414	1.336	0.979	6.388	5.193	36.49	9.789	985.3	*2034.1*	
88	0.331	0.960	3.881	2.151		1.648	0.960	1.887	21.70	0.655	587.2	8.500	509.7	14.54	*1743.3*		*1294.3*		2.547	2.464	1.398	1.161	6.952	6.970	37.69	12.32	*3564.2*		
96	0.338	0.999	4.414	2.012		1.546	0.705	1.836	21.81	0.579	705.8	*602.1*		14.39	*2001.7*		*1596.4*		2.408	2.352	1.440	1.114	7.209	5.121	37.66	*3930.4*			
104	0.247	1.032	3.956	1.790		1.297	0.599	1.856	21.43	*1572.9*				14.84	*2373.3*		*2109.7*		2.067	2.529	1.476	1.133	7.306	*4555.6*					
112	0.365	1.018	4.366	2.043		1.577	0.756	1.897	21.47	*1769.5*				14.50	*2749.3*		*2436.9*		2.356	2.451	1.483	1.121	7.328	*5073.7*					
120	0.397	1.015	4.387	2.135		1.618	0.819	1.813	22.59	*2172.4*				*6314.0*					2.527	2.417	*5706.2*								
128	0.364	1.022	4.795	2.060		1.621	0.742	1.835	21.92	*2562.3*				*13545.9*															
136	0.417	1.109	4.589	2.193		1.673	0.823	2.052	22.85	*3073.0*				*14954.1*															
144	0.412	1.047	4.443	2.089		1.588	0.770	1.853	*20323.9*																				
152	0.456	1.034	4.575	2.078		1.678	0.826	1.843	*22055.2*																				
160	0.445	1.076	4.766	2.148		1.683	0.833	*24631.9*																					
168	0.442	1.152	4.818	2.143		1.644	0.876	*26608.1*																					
176	0.472	1.073	4.869	2.166		1.668	*28730.5*																						
184	0.446	1.149	97.72	2.217		*30155.5*																							
192	0.471	1.101	98.33	2.223	147.1	*30965.2*																							
200	0.335	1.172	99.60	2.068	459.6	*32415.2*																							
208	0.375	1.028	101.6	26.81	*33929.8*																								
216	0.370	1.209	109.2	26.82	*35362.6*																								

Comparing Figure 3a,b, it is observed that most corrosion pits were formed near the specimen surface within the first 8 hours of immersion. The exceptions include pits 22 and 27 which were formed after 16 h, and pit 28 after 64 h (all near the surface). Figure 3 also indicates a degree of correlation between corrosion pit initiation sites and near‐surface porosity with 94% of pits being identified with void structures present near the surface of the sample prior to corrosion (Table 2; compare Figure 2a,b). Online interactive Web3D visualizations of the pits after different immersion times are available in a supplementary dataset.^[^ ^41^ ^]^

Figure 3 shows that the sizes of some pits remain virtually unchanged with time after initiation, such as those labeled 1–17. Other pits, particularly those near the top and bottom of the specimen, continued to grow with time. They grew preferentially within the powder‐bed plane compared to the build direction, as shown in Figure 3d,e. This is because LOF structures tend to form in single spatter events on the same plane as the powder bed.^[^ ^4^ ^]^ The thickness of the spatter particles (typically > 100 µm) means that the LOF structures may extend over several powder bed layers which are 30 µm thick (Table 1).^[^ ^4^ ^]^ Thus, during spatter, the spatter balls of molten metal (316L SS in this case) have an influence on the density of layers subsequently formed around and above them. The original anisotropically distributed LOF pore structure is particularly visible in Figure 3a where the pore dimensions are larger in the horizontal (powder‐bed plane) direction than in the vertical (building) direction.

Figure 4 shows the LOF pore clusters prior to exposure to the FeCl_3_ solution and LOF pores and pits after exposure for the low‐density 316L SS specimen. Most of the corrosion pits appeared after 8 h of immersion, with pits 14 and 27 as exceptions. In this case the original LOF pores and corrosion pits showed greater anisotropy in the build plane (parallel to cylindrical axial) than the mid‐density sample. 100% of pits were associated with preexisting void structures. Web3D visualizations of the pits after different immersion times are available in a separate supplementary dataset.^[^ ^42^ ^]^

### 3.2 Total Pit Volume Growth

**Figure 5:**
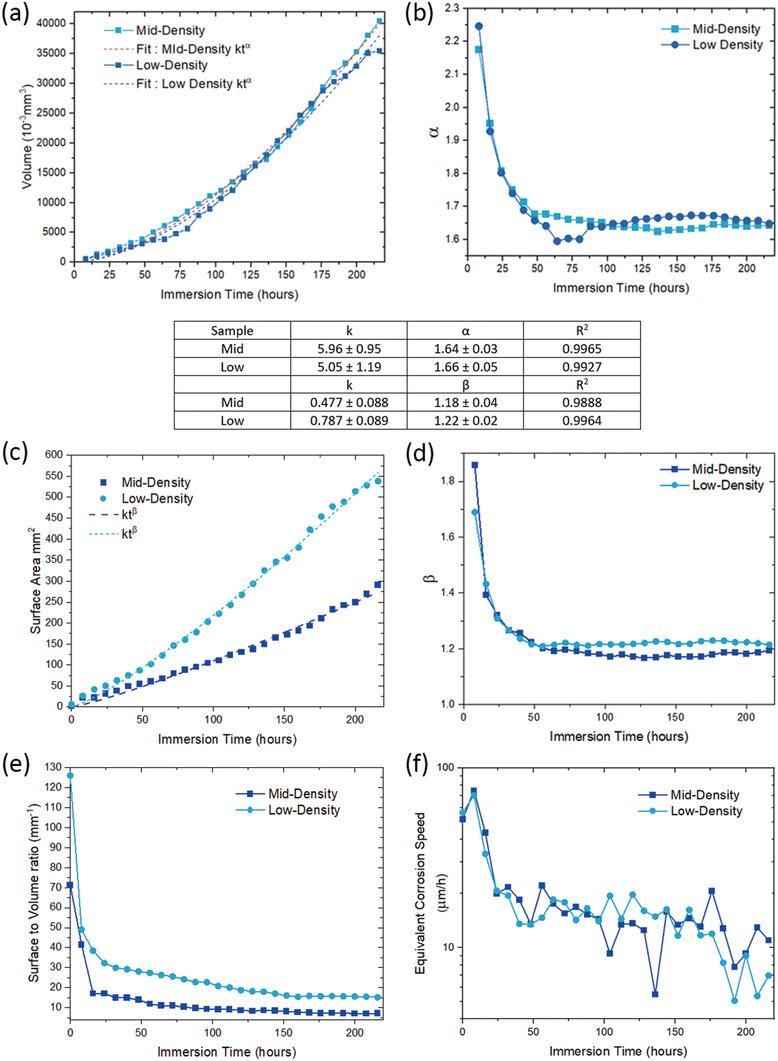
Whole sample corrosion kinetics for the low‐ and mid‐density LPBF 316L SS specimens as a function of immersion time. a) Total pit volume *V*_f_, b) *α*‐values, c) total pit surface area, d) *β*‐values, e) total pit surface to volume ratio, and f) equivalent corrosion speed *d*∅/*dt*.

The total corrosion pit volumes *V_f_* of each specimen were calculated by summing over the labeled pits in Tables 2 and 3. **Figure** **5**a indicates that the total corrosion pit volume can be well fitted using power‐law curves (*V_f_ = kt^*α*^*) versus the immersion time *t* with *R* ^2^ values of 0.996 and 0.992 for the mid‐ and low‐density specimens, respectively. The *α*‐values were 1.66 and 1.64 for the mid‐ and low‐density specimens, respectively. Direct fitting provides information on the global growth over the whole experiment. In particular the index in this type of power law (*α* in this case) is often used to describe different kinetic growth for depth or width of pits with *α* = 0.5 indicating diffusion limited depth growth in pits and *α* = 1 for width growth under lacy pit covers. The “instantaneous” *α* values can be obtained from

(1) $$\ln\left(V_{f}\right)=\ln\left(k\right)+\alpha \ln\left(t\right)$$

where *k* is obtained by fitting *V_f_ = kt^*α*^*. This “instantaneous” *α* is an indicator of the difference between the fitted curve in Figure 5a and the data. It can be seen in Figure 5b that the biggest difference is in the early stages of pit growth where *α* is in the range 2.15–2.25 where the curve fit is poorest, but thereafter decreases to values fluctuating between 1.6 and 1.7 reflecting different pitting behavior in the early stages of pit growth. The surface area of the pit can be deduced in a similar fashion by fitting *S_f_ = kt^*β*^* with the data presented in Figure 5c,d for surface area and beta, respectively. Figure 5c shows that the low‐density sample develops a much higher surface area during the latter stages of corrosion than the mid‐density sample. In Figure 5d, *β* for both samples is generally between 1.0 and 1.2 indicating mostly linear growth with time. This type of behavior suggests that pit growth is under diffusion control through an Fe‐rich salt film.^[^ ^28^, ^43^ ^]^ Figure 5e shows that the total surface area to volume ratio for the low‐density specimen is consistently higher than that for the mid‐density specimen due to the elongated corrosion pit shape in the low‐density specimen (c.f. Figures 3 and 4).

The equivalent spherical diameter of the total pit volume was estimated using ∅ = (6*V_f_*/*π*)^1/3^. The equivalent corrosion speed *d*∅/*dt* is shown in Figure 5f. At the earliest times, the equivalent corrosion speed was about 77 and 70 µm h^−1^ for the mid‐ and low‐density specimens, respectively, and slowed to as low as 7.7 and 5.0 µm h^−1^ at the longest times.

Figure 5 shows that, up to about 50 h of immersion, both specimens demonstrated a mix of volume and surface area limited growth. Between 50 h and 175 h pit growth was approximately a surface‐area‐limited corrosion behavior with *α* ≈ 1.65 and 1.0 ≤ *β* ≤ 1.2. As seen below, these time regimes coincide with individual, competitive and dominant pit growth. Based on the d*ϕ*/d*t* values, it also indicates that prior to competitive growth, individual pit growth is around ten times faster compared to when they enter the competitive growth regime. In previous studies, linear growth of the (pit depth)^2^ with time has been attributed to diffusion‐controlled corrosion under a salt film.^[^ ^28^, ^29^, ^44^ ^]^

### 3.3 Corrosion Initiation and Early Propagation Sites

**Figure 6:**
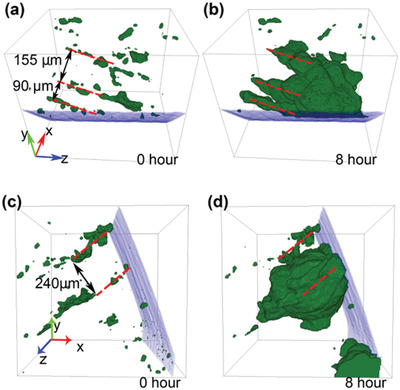
Close‐up views of corrosion pit formation in the low‐density specimen before (left) and after 8 h immersion (right). Pits are displayed as green and the surface of the specimen is displayed in blue. a,b) Original pores near before corrosion and pit 12 (grid: 150 × 150 × 150 voxels (600 × 600 × 720 µm^3^)). c,d) Original pores before corrosion and pit 15 (grid: of 200 × 200 × 170 voxels (800 × 800 × 680 µm^3^)).

It is clear from Figures 3 and 4 that corrosion initiation and early propagation are correlated with preexisting pore structures (97%–100%). As an example, **Figure** **6** shows a magnified view of the microstructures near pits 12 and 15 (low‐density specimen) before and after 8 h of immersion. In both cases these structures are where LOF hatch boundaries (LOF‐HB) intersect the exterior of the specimen. Comparing original pores and pit 12, it was observed that corrosion propagation tends to follow the original LOF‐HB (red dashed lines in Figure 6a,b). The separation of these structures is 90 µm (close to hatch width) and 155 µm (≈1.7 times the hatch width). The distance propagated in the 8 h equates to a d*ϕ*/d*t* ≈ 72 µm h^−1^. For pit 15, a similar behavior is observed when comparing Figure 6c with Figure 6d where the greatest propagation was along the original LOF‐HB separated by about 240 µm (≈3 times the hatch width). The growth along these hatch structures shows protuberances in the hatch direction where corrosion growth is most rapid. The distance propagated in the 8 h equates to d*ϕ*/d*t* ≈ 60 µm h^−1^. In both of these cases the front velocity is in good agreement with the global velocity presented in Figure 5f. These protuberances are a signature of preferential pit growth through LOF structures throughout this study.

### 3.4 Local Pit Propagation at Longer Times

**Figure 7:**
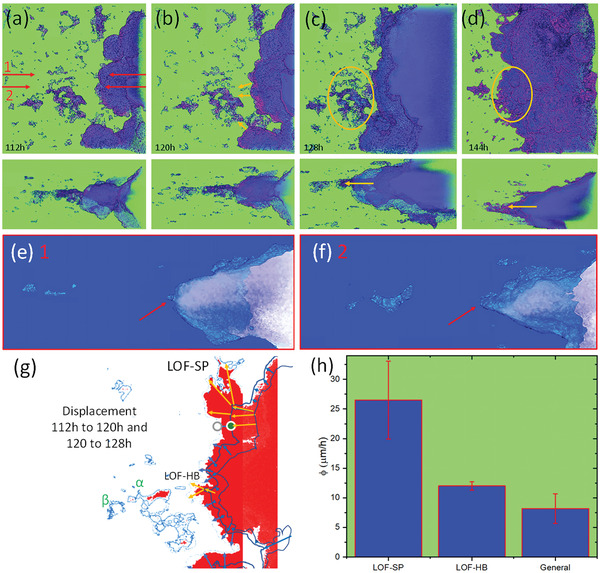
a–d) Top and side views of corrosion fronts at 112, 120, 128, and 144 h, respectively. The sets of reds arrows labeled “1” and “2” in (a) are where the sections shown in (e) and (f) are located. Orange arrows in (b) show LOF structures into which corrosion is propagating. Orange ellipses in (c) and (d) show major LOF‐SP structures which have been consumed by corrosion. g) Distance propagated by corrosion between 112 and 120 h as well as 120 and 128 h. h) d*ϕ*/d*t* for LOF structures and the general matrix.

Propagation at later stages is also influenced by the pore structures. For example, **Figure** **7** presents reconstructions from the same region of the combined pit 19 and 20 for times from 112 to 144 h. LOF structures and the metal/pit interface are displayed in purple. Figure 7a shows the pit front on the left and LOF structures which will be consumed in subsequent time steps shown in the center and right. Two pairs of red arrows in Figure 7a indicate where slices have been taken through the pit and are presented in Figure 7e,f, respectively. In Figure 7e, there is a small feature which is an LOF feature protruding from the pit front which aligns with the LOF structures further to the left (ahead of the pit). In Figure 7f, there is a larger feature which is again aligned to the LOF structures to the left (ahead of the pit). Figure 7b shows the direction of propagation of pitting indicated by the orange arrows. The LOF structures highlighted by the orange ellipse (Figure 7c) have been completely consumed by 144 h (Figure 7d). Figure 7g also shows the position of the corrosion front in blue at 112 and 120 h superimposed onto the corrosion front at 128 h (solid red). Detailed measurement of the displacement of the corrosion front propagation was made providing an estimate of how far the front progressed in the previous 8 h. These measurements were divided into etching of the matrix (blue arrows) as well as LOF structures (orange arrows). They were then used to calculate the local velocity of the corrosion front as detailed below. The velocity of the corrosion front d*ϕ*/d*t* is depicted in Figure 7h. The velocity through the LOF spatter particle (LOF‐SP) structures is around 26 µm h^−1^ and through the LOF‐HB structures is ≈12 µm h^−1^ which is around 3.5 and 1.6 times that through the matrix. Given that this corrosion front is moving through the specimen between 112 and 144 h, these figures are in good agreement with the global figures presented in Figure 5f. The greater susceptibility of these LOF structures probably results from a range of characteristics not seen in the general matrix including defect structures, surficial oxides and voids.^[^ ^4^ ^]^ Like the 8 h pit propagation data, accelerated corrosion in LOF structures manifested as protrusions in the 3D reconstructions of the voids described below.

### 3.5 Analysis of Individual Pit Growth Kinetics

In Table 2, gray backgrounds represent merged pits and those highlighted in blue (pits 1–18 and 21–26) have ceased to grow at the time at which they are highlighted. These are generally in the vicinity of 1 × 10^−3^ to 20 × 10^−3^ mm^3^ with the vast majority being at the lower end of the range. Their size is much larger than metastable pitting events and they should be classified as repassivated/deactivated pits many of which end up being consumed by larger pits. The rest of the pits had volumes that increased with the time. In Table 2, pits 19 and 20 at the top were merged after 16 h and continued to grow. Pits 28–33 near the bottom merged into one pit after 64 h of immersion, merging with pit 27 after 152 h, then extended towards the top and merged with the pits near the middle of the specimen.

**Figure 8:**
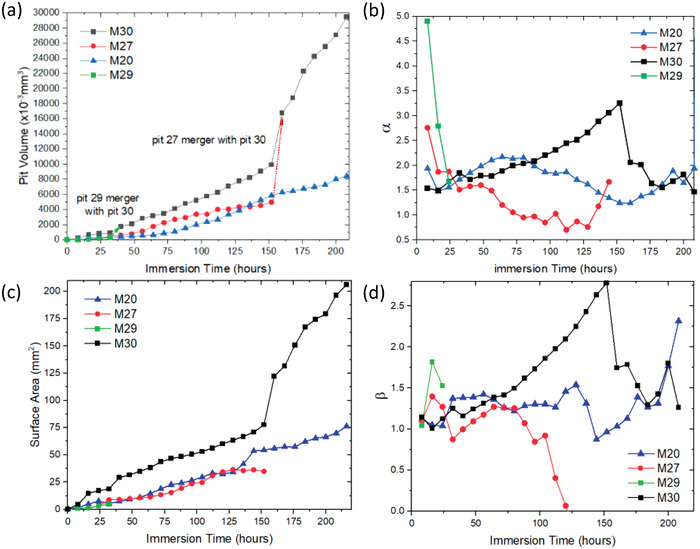
The pit volumes, surface areas, and their power‐law exponents for time‐varying corrosion pits for the mid‐density specimen. a) Pit volume and b) *α* versus immersion time. c) Pit surface area and d) *β* versus immersion time.

The corrosion pit volumes and surface areas versus time for four pits are shown in **Figure** **8**a,c, respectively. The power‐law exponent values for pit volume and surface areas were estimated as *α* = ln(*V*/*V_max_*)/ln (*t*/*t_max_*) and *β* = ln(*S*/*S_max_*)/ln(*t*/*t_max_*), respectively, as shown in Figure 8b,d. Interpretation of the data using power laws is divided into two regimes: i) prior to pit mergers and ii) post pit mergers (particularly large pit mergers). In the latter case, the large increase in volume cannot be fitted with a single power law. Prior to pit mergers, the pit volumes and surface areas showed an approximate power‐law dependence with time. For large pits, e.g., pits 20, 27, and 30, even though the volumes for these pits are increasing, the *α* values show no consistent trend suggesting that individual pit kinetics vary considerably with values between 1.5 and 2.2 and *β* ≃ 1.2. This suggests that the corrosion pit was growing slightly faster than via a diffusion‐controlled process and perhaps, given *β*, strongly under the influence of a surface‐area controlled process, i.e., the corrosion proceeds via processes occurring at the interface of the metal with the internal electrolyte, i.e., via salt and passive film formation. To briefly put some context to these values it is worth noting that many pit growth studies^[^ ^28^, ^43^ ^]^ report only changes in *one dimension* of either pit width or depth with growth rates following *t* and *t* ^0.5^, respectively, these need to be combined to directly compare to overall pit *volume* growth of *t* ^1.5^. The linear dependence of width on time reflects the development of lacy pit covers where the internal electrolyte under the surface regularly perforates the surface, leading to release of anolyte and change in the salt film which reactivates the pitting process. Pit depth growth based on *t* ^0.5^ is usually interpreted as diffusion limited with a pit volume growth rate of *t* ^1.5^.

**Figure 9:**
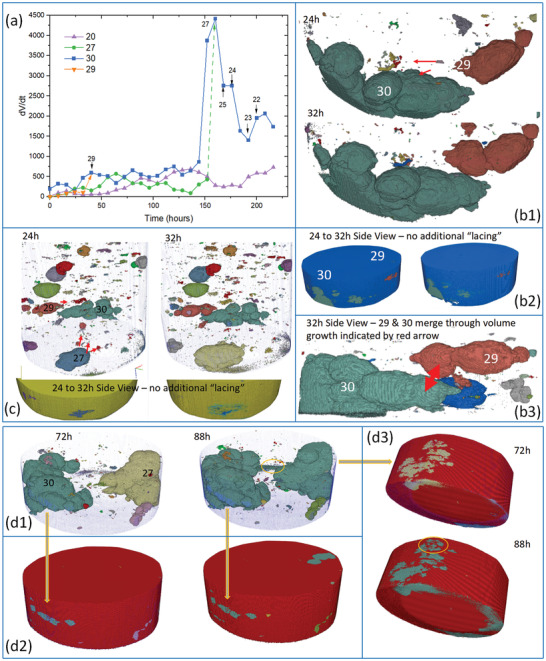
a) Pit volume growth rate (d*V*/d*t*) versus time for pits 20, 27, 29, and 30 [labeled in (b)]. The small black numbers with arrows pointing toward the curve for pit 30 indicate where other pits are subsumed. b) Detail of growth of pits 29 and 30 between 24 and 32 h. Pit 30 shows no change whereas pit 29 develops along protuberances around the top and bottom red arrow and grows outward. c) DCM reconstruction showing pits 27, 29, and 30 at 24 and 32 h (left and right, respectively) as well as the entrance points on the exterior of the sample below the reconstructions. d) Growth of pits 27 and 30 from 72 to 88 h.

The rate of growth in the pit volume (d*V*/d*t*) was further examined for a number of pits as displayed in **Figure** **9**a where it can be seen that pits show periods of accelerated growth and slower growth. Close inspection of the trends showed some interplay between the pit growth rates, i.e., whenever one pit shows a greater growth rate than other pits show slower growth rates. This is most evident for pit 30 which is a combination of pits labeled 28–33 in Figure 3. It goes through several small growth spurts but when each of these growth spurts is finished, other pits display growth spurts. This happens around 24 h when pit 30 shows a small decrease in growth rate but 27 and 29 display a modest increase in growth. In the case of pit 29 (Figure 9b1), it appears to display growth in the hatch direction indicated by the red arrows followed by the generation of an additional lobe of corrosion with further growth along a hatch feature as indicated by the protuberance. A side‐on view of these two pits shows that they are propagating in the build plane (Figure 9b3).

**Figure 10:**
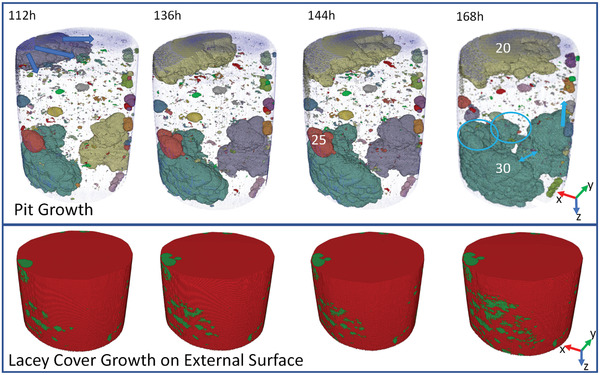
Top void reconstructions as represented by cluster analysis for 112–168 h and bottom: entry points on the external surface as indicated by growth of lacy covers for the same times. The blue ellipse in the void reconstruction for 144 h represents where pits 27 and 30 merge and those at 168 h indicate where rapid growth has occurred. The blue arrows indicate the direction of growth on the other side of the pit.

Between 24 and 32 h the growth in pit 29 shows only internal growth (*α* decreases from 4.9 to 1.67) since no new pit openings are developed on the sample surface (Figure 9b2) and appears to happen at the expense of growth of pit 30 which shows no changes during this period. Pit 29 and 30 eventually merge by volume growth at the location indicated by the double ended red arrow in Figure 9b3. Pit 27's growth over the same period coincides with a lower *α* (1.8 → 1.5) and shows some lacy cover development (Figure 9c). The growth rate of pit 30 increases a little between 72 and 88 h with *α* ≈ 2 and no lacy cover development (Figure 9d2). Over the same period, Pit 27's growth rate decreases modestly but there is still some lacy cover development (orange ellipse in Figure 9d3) and *α* ≈ 1. Pit 27 grows from around 136 h and its *α* value increases from 0.76 to 1.67. Pit 30 shows a huge increase in growth rate (*α* ≈ 2.3). The growth is largely upwards and includes incorporation of pit 25 plus a second node indicated in **Figure** **10** (blue ellipse: 168 h) and the right‐hand side of pit 30 is also growing upwards as well as across the sample. Both sides of pit 30 show increased area of lacy cover suggesting active undersurface growth. Pit 20 also begins to grow again with an *α* (1.2 → 1.9). Between 144 and 168 h the growth rate of Pit 30 has dropped off, extensive lacy cover has developed and *α* has dropped from 3.3 to about 1.5. The interplay between growth rates of different pits continues until around 150 h where pit 30 becomes the dominant pit by consumption of other pits (the pit number and the time it was consumed are indicated in Figure 9a). This interplay suggests some limitation of the available cathodic current globally for the sample. Both these pits have connection to the external electrolyte so the merging of these features may lead to convection effects within the new joint pit volume.

The same analysis as detailed above can be applied to the low‐density specimen for pits labeled in Figure 4b. Table 3 shows that, after 216 h of immersion, except for pits 1 → 4, all other corrosion pits merged together. Similar to the case with the mid‐density specimen, some pits increased in size with time and propagated in the build plane which is parallel to the vertical direction in Figure 4. These include pits 10, 12, 15, 17, 26, and 28. Other pits had ceased to grow before they merged with growing pits (blue highlight, Table 3) and had similar size distributions to the mid‐density sample.

**Figure 11:**
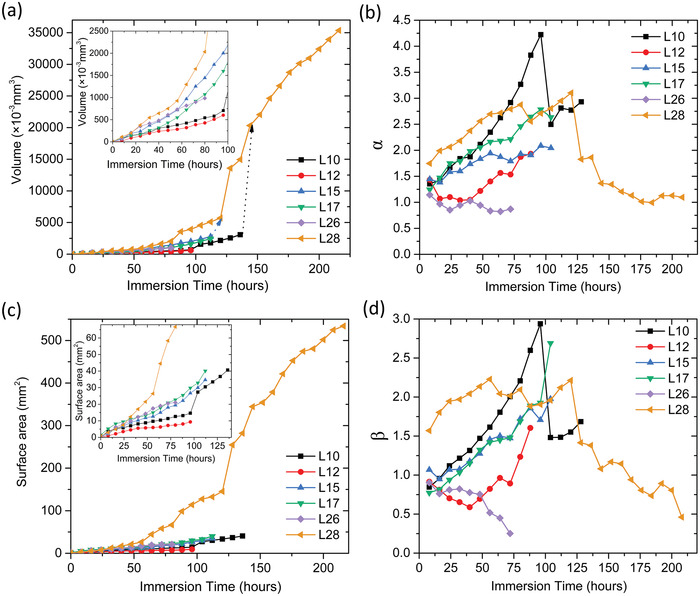
The volumes and power‐law exponents versus time for time‐varying corrosion pits for the low‐density specimen. a) Pit volume, b) volume exponent *α* = ln(*V*/*V_max_*)/ln (*t*/*t_max_*), and c) pit surface area, and d) surface area exponent *β* = ln(*S*/*S_max_*)/ln(*t*/*t_max_*).

The increase in volume and surface area of pits that grew with time are shown in **Figure** **11**. Pits 14 and 15, and pits 16 and 17 merged forming single pits after 40 and 24 h of immersion and were relabeled as pits 15 and 17, respectively. Similar to the mid‐density case, pit volumes and surface areas versus time (Figure 11a,c) both showed an approximate power‐law behavior with *α* and *β* values shown in Figure 11c,d. The *α*‐value for the combined pit 15 and the value for pit 17 were distributed around a value of *α* ≃ 2. Their *β*‐values are between 1.5 and 2.1, that is, the pit surface area was growing faster than time. The *α*‐value for the pit 28 and pit 10 values were increasing to values of 3.0 and well over 3.0, respectively. This latter data are based on merged pits hence including merged pits.

**Figure 12:**
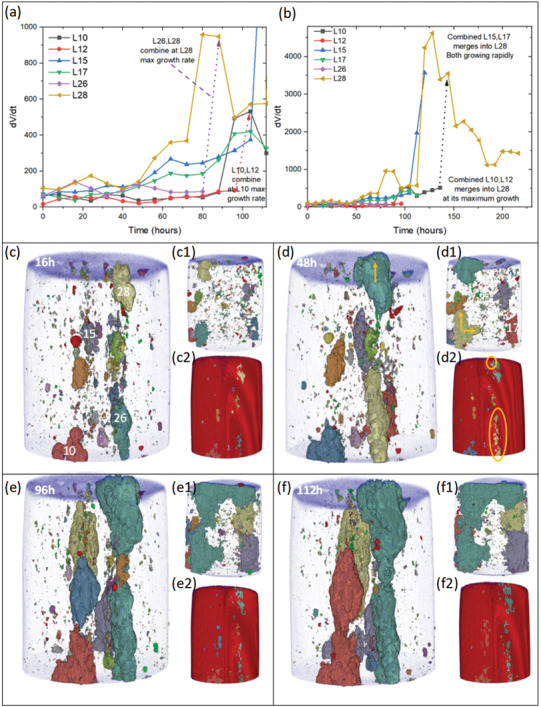
a,b) Pit volume growth rate (d*V*/d*t*) for pits 10, 12, 15, 17, 26, and 28 (labeled in Figure 4b) versus time. c–f) DCM reconstruction showing pit reconstructions for 16, 48, 96, and 112 h, respectively, as well as the entrance points on the exterior of the sample.

The rate of change in volume growth is depicted in **Figure** **12**a,b. In Figure 12a the early stages of pit growth are shown for pits 10, 12, 15, 17, 26, and 28. To begin, pit 26 showed some accelerated growth up to 16 h, but its growth slowed as pit 28 became active up to 24 h. The growth of pits 10 and 26 is largely subsurface with extensive lacy cover development and *α* ≈ 1. The pit growth rate for Pit 28 then slowed slightly as pit 10 and 15 began to accelerate up to 40 h where pit 10 trailed off but pit 15 continued to grow. During this period pit 10 was penetrating into the sample (*α* ≈ 1.5 →1.9) where pit 15 was a mixture of mostly penetration into the sample and some subsurface (lacy cover) growth (*α* ≈ 1.5→1.8). So by 48 h Figure 12d1,d2 shows significant extension in the build plane (Figure 12d) with pit 28 mostly penetrating into the sample (*α* ≈ 2.4 →2.8) (Figure 12d, 1) and pit 26 mostly growing via a lacy cover (*α* ≈ 1) (orange ellipse in Figure 12d2). Examination of Figure 12d1 shows that the volume and surface growth of pit 28 (penetrating into the sample along the build plane) is greater than pit 26 which has its major component as lacy cover propagation (*α* ≈ 1). On the other hand, *β* is between 1 and 1.5 for pit 28 compared to *β* ≈ 0.75 for pit 26 suggesting that corrosion propagation along the build plane via LOF structures creates a larger surface‐to‐volume ratio. Pit 24 grew up to 40 h then stabilized and was consumed in the growth spurt of the combined pit 26 and 28 at 112 h (Figure 12f1,f2). The pit growth rate of pit 26 above 48 h slowed but was characterized by growth underneath the surface in the build plane created a lacy cover on its surface as well as into the build plane away from the surface (Figure 12). The growth of these two pits then fell off a little as pits 15 and 17 on the opposite side of the sample increased their growth rate between 48 and 72 h (*α* ≈ 1.75 and *α* ≈ 2.0, respectively). The main area of growth for these two pits was into the sample along the build plane with only small changes due to lacy cover growth. These metrics indicate that growth into and along the build plane is faster than growth achieved via a lacy cover mechanism. The interplay is more evident between 72 and 112 h where pit 28 shows some rapid growth toward pit 26 and had combined with it at 88 h where its growth rate dropped off but pit 10's rate increased to the point where it had combined with pit 12 by 96 h (Figure 12e1,e2). The combined pits (labeled pit 10 in Figure 12a) show a growth spurt up to 112 h where the combined pit 26/28 shows a huge increase to become the dominant pit and pit 10/12 drops off eventually combining with pit 28 at 144 h. It can be seen in Figure 12e1,f1 that pit 28/26 shows very little evidence of pit growth from 96 to 112 h.

## 4 Discussion

The critical role of LOF pores in localized corrosion in LPBF 316L SS has been reported in a recent study by the authors^[^ ^19^ ^]^ where pitting initiation was found to be closely associated with porosity such as LOF pores. Close inspection of a single LOF pore in a higher density (≈99.1%) specimen showed that localized corrosion tended to initiate and develop preferentially from LOF pores that intersected the outer surface of the specimen. However, the detailed mechanism and processes whereby pores affect the kinetics of pit volume growth remained unclear and therefore highlight the need for this study. Since that early study, the authors have classified several different kinds of LOF pore structures according to their geometry and formation mechanism.^[^ ^4^ ^]^

In this study, pit initiation at LOF structures was again evaluated in situ, with Figures 2, 3, 4 indicating close to 100% correlation between corrosion pit initiation sites and near‐surface porosity. Moreover, pitting propagation was influenced by LOF geometry and spatial distribution with accelerated propagation along these structures. The emerging picture is a combination of mechanisms contributing to pit growth in the LPBF SS316 studied here including:

i. Three growth periods
a. an initial, rapid growth period for pits initiating from LOF structures connected to the external surface;
b. a period of interplay between pits during growth, presumably competing for enough cathode area to support anodic dissolution;
c. dominant pit growth.
ii. A new mechanism where penetration into the material is facilitated by corrosion of LOF structures. This leads to deep pits.
iii. Subsurface growth characterized by lacy covers is indicative of undersurface corrosion perforating the metal surface, potentially expelling internal electrolyte and probably disrupting passive or salt film formation.
iv. Merging of pits where increased volume and new exits from pits may cause electrolyte convection leading to electrolyte expulsion/dilution.

### 4.1 Early Pit Propagation

**Figure 13:**
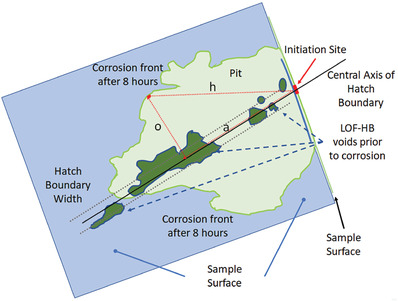
Schematic of pit shown in Figure 6d with overlay of LOF structures prior to corrosion taken from Figure 6c. Blue indicates the interior of the sample, and light green the pit outline and void. The red arrows are a construct to show possible pit propagation from the initiation site to an arbitrary point on the surface of the pit. It should be noted that path “a” has a mixture of LOF structures and matrix along its length. The derivation of the velocity for both types of LOF structures in Figure 7h takes into account etching through matrix as well as LOF structures and so the velocity itself is an average over both structures exists along path “a”.

Pit initiation and propagation were clearly influenced by LOF structures. The method of propagation appears to be via electrolyte ingress into LOF pores followed by etching of the surrounding matrix (c.f. Figure 6). Clearly acidification of the FeCl_3_ electrolyte in LOF pores is possible since they are small occluded volumes where the pH can decrease to as low as pH 0.5 compared to pH 2 of the external electrolyte thus favoring the establishment the critical pitting/crevice conditions, where the acidification accelerates the corrosion propagation.^[^ ^45^ ^]^ Propagation was often directed toward neighboring LOF pores in many instances apparently guided by LOF‐HBs. An example of penetration of LOF‐HB structure can be seen in **Figure** **13** based on the pit shown in Figure 6c,d. Considering the triangle with sides labeled “a”, “o”, and “h”, while path “h” may be the shortest path from the initiation site to a point on the corrosion front, corrosion can reach the same place in a shorter time if the velocity of the pit front is faster along path “a”. Indeed, the velocity along “a” would need to be only 1.65 times the velocity along “h” (assuming the velocity along “o” is the same as “h”) to reach the same point at the same time. Given that the velocity along LOF‐SP features is considerably higher (>3) and for LOF‐HB features is in the vicinity of 1.65 (Figure 7h) then this mode of preferred propagation along the LOF‐HB is quite feasible. Thus, pitting seems to be a two‐stage process: i) a corroded “tunnel” develops along the LOF structure followed by ii) the corrosion of the surrounding matrix creating a corrosion “chamber.” This “tunnel and chamber” method of growth is seen repeatedly for a number of pits on both specimens studied here and is responsible for pit growth as well as pit merger. It should lead to higher surface‐to‐volume ratios, which is consistent with the greater surface‐to‐volume ratio for the low‐density versus the mid‐density specimens shown in Figure 5e. Moreover, because pit growth tends to follow these LOF structures and these structures tend to occur in the build/powder bed plane, this leads to rapid penetration into the sample initially reducing the likelihood for lacy cover development as observed in conventional 316L SS. In some respects this model has similarities to the morphological aspect of stress corrosion cracking where crack growth runs ahead of pit growth. It is expected here that the LOF structure is a static structure but it does allow for ingress of the electrolyte ahead of the main pit.^[^ ^46^ ^]^ This growth seems to be characterized by higher *α* values. It is also worth noting that the more rapid attack along LOF‐HB structures suggests a greater susceptibility of the hatch boundary which warrants further investigation of the microstructure at these boundaries.

### 4.2 Global Pit Growth

It is interesting to note, that from the outset the overall corrosion of the samples is “well behaved” as described by a *V* = *kt* ^1.66^ behavior (Figure 5a) even though individual pits had *α* values that varied widely over time. This suggests that individual pit growth rates were controlled by global aspects of the sample, most likely the available cathodic current. This is consistent with the interplay between pit growth observed in Figures 9 and 12. This period of interplay continues up to the time when a dominant pit drives most pit growth and involves merger with other pits.

**Figure 14:**
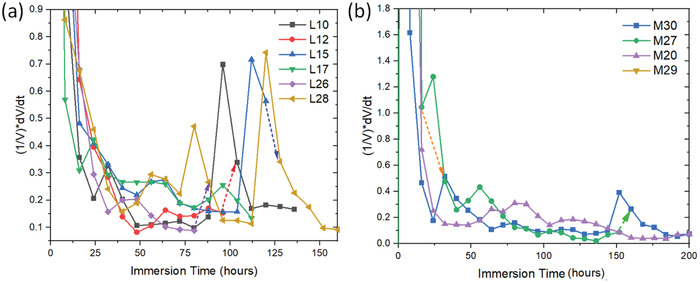
Growth rate per unit volume of pits: a) pits in low and (b) medium density samples.

On the aspect of growth rate, the initial global growth rate is in the vicinity of 70 µm h^−1^. This value is of a similar order to that reported by Heurtault et al.^[^ ^47^ ^]^ of around 140 µm h^−1^ in aggressive 0.5 m H_2_SO_4_/3 m NaCl solution for pit depth growth. In their case, they demonstrated that pit depth grew under diffusion control. Here, however, the corrosion rate of individual pits varies considerably with time, but the results indicate that the growth is controlled by corrosion reactions of different internal structures of the sample appearing within the pits. Overall, these results suggest a significant role for diffusion‐controlled growth beneath salt film established at the metal–electrolyte interface. There is also evidence to suggest that some growth may occur without the salt film since some local growth suggests *β*‐values higher than 1. Control of corrosion at the pit surface in these conditions is usually dictated by salt film formation. One potential cause for salt film breakdown, may be the consumption of smaller pits by larger pits. It is proposed that this consumption process results in a dynamic redistribution of internal electrolyte within the pit. For example, the growth rate per unit volume of any individual pit is shown for the low and medium density samples in **Figure** **14**a,b respectively. It can be seen in the low‐density sample that pit 10 goes through two growth spurts at around 75 and 125 h immersion. Every time pit growth results in consumption of adjacent pits (pit 26 at 75 h and pit 15 at 125 h), the growth rate decreases significantly. It would be reasonable to assume that when pit 10 is going through a growth spurt, it is the anodic head that eventually eats into the adjacent pit. As a consequence, the anolyte solution is dissipated into the new pit resulting in a loss of activity in pit 10. There are numerous more examples of this behavior in Figure 14 for the low and medium density samples.

### 4.3 Lacy Covers

While the tunnel and chamber model favors pit propagation into the sample, particularly in the build plane, there is still the possibility for the development of lacy covers when much of the local internal volume has been consumed. This is also evident in Figure 2c,f where, after corrosion, the outer shell remains intact retaining a thin layer of the original sample surface (Figure 1a) as well as the mouths of corrosion pits at completion of the experiments. Such pitting morphology is consistent with conventional pitting processes: local breakdown of the passive layer by chloride ions and the formation of passive cathodic regions and active anodic areas, although the corrosion features are somewhat unusual in this instance. Oxygen reduction should occur just outside the surface openings, resulting in a higher pH on the local surface opening area. Such a local higher pH would make the thin layer of metal near the sample surface cathodic, i.e., cathodically protected, thus remaining intact.

It should be noted that the periodic removal of the LPBF specimens from the corrosive media for X‐ray CT testing could perturb corrosion processes to some extent, although some corrosive solutions would remain inside the porosities of these specimens and corrosion processes are expected to continue throughout the whole testing period. During X‐ray CT imaging, the corrosion process was slowed down as the corrosion media diffusion between pits and bulk corrosion solution was interrupted. Potential improvements to the testing procedure will be explored in future investigations to minimize the disruption of corrosion processes. Future corrosion tests will also be run in other corrosion media such as seawater in additional to the highly aggressive ferric chloride solution.

## 5 Conclusion

The immersion corrosion behavior of LPBF 316L SS specimens was studied using in situ X‐ray CT to investigate the pitting initiation, propagation, and growth behavior and kinetics and the effects of distinctive microstructural features. Experiments have been carried out to demonstrate the capabilities of the in‐situ X‐ray CT in probing the mechanism and kinetics of localized corrosion, to identify the pitting growth mechanisms of LPBF stainless steel that are different from the conventional stainless steels, and to reveal the pit growth kinetics through 3D in situ X‐ray CT data analysis. More specifically, main findings and conclusions made from this research are as follows:

1. It was confirmed that the corrosion pit initiation was correlated with the original LOF porosity distribution on the surface of the specimens.
2. Pitting propagation occurred more rapidly through LOF structures then involved etching of the matrix. A “tunnel and chamber” model is proposed where there is rapid penetration in the build plane through both LOF‐HB and LOF‐SP.
3. Pitting appeared to occur in three different regimes, initial individual pit formation, followed by competitive pit propagation, leading to the growth of a dominant pit. Some pits only grow during the initial individual pit growth period.
4. The global corrosion pit size versus time showed an approximate power‐law behavior, but individual pit growth appeared to be modulated by local kinetics related to the internal microstructure. There was a linear growth of the surface area with time, suggesting that diffusion control through a salt film was the dominant form of pit growth. However, for individual pits there was considerable variation from the global growth characteristics.
5. The influence of LOF structures could be detected in the trends of the S/V ratio for pitting comparing the mid‐ and low‐density specimens. A much high S/V ratio was observed during the period of competitive growth in the low‐density sample which had a higher number of LOF structures.

## Conflict of Interest

The authors declare no conflict of interest.

## Data Availability

The data that support the findings of this study are openly available in CSIRO Data Collection at https://doi.org/10.25919/4ksz‐vj58.
